# Soil total carbon and nitrogen and crop yields after eight years of tillage, crop rotation, and cultural practice

**DOI:** 10.1016/j.heliyon.2017.e00481

**Published:** 2018-01-11

**Authors:** Upendra M. Sainju, Andrew W. Lenssen, Brett L. Allen, William B. Stevens, Jalal D. Jabro

**Affiliations:** a USDA, Agricultural Research Service, Northern Plains Agricultural Research Laboratory, 1500 North Central Avenue, Sidney, Montana 59270, USA; b Department of Agronomy, Iowa State University, Ames, IA 50011, USA

**Keywords:** Agriculture

## Abstract

Information on the long-term effect of management practices on soil C and N stocks is lacking. An experiment was conducted from 2004 to 2011 in the northern Great Plains, USA to examine the effects of tillage, crop rotation, and cultural practice on annualized crop residue (stems + leaves) returned to the soil and grain yield, and soil total C (STC) and total N (STN) stocks at the 0–120 cm depth. Tillage practices were no-tillage (NT) and conventional tillage (CT) and crop rotations were continuous spring wheat (*Triticum aestivum* L.) (CW), spring wheat-pea (*Pisum sativum* L.) (W-P), spring wheat-barley (*Hordeum vulgaris* L.) hay-pea (W-B-P), and spring wheat-barley hay-corn (*Zea mays* L.)-pea (W-B-C-P). Cultural practices were traditional (conventional seed rates and plant spacing, conventional planting date, broadcast N fertilization, and reduced stubble height) and improved (variable seed rates and plant spacing, delayed planting, banded N fertilization, and increased stubble height). Crop residue and grain yield were greater with CW and W-P than W-B-P and grain yield was greater with the traditional than the improved practice. The STC at 10–20 and 90–120 cm was greater with CW or W-P than other crop rotations in CT and greater with CW than W-B-P in NT. The STN at 20–40 cm was greater with W-P than CW and W-B-P in CT. With NT and the improved cultural practice, STN at 0–5, 5–10, 20–40, and 60–90 cm was greater with W-P and W-B-C-P than other crop rotations. The STN at 0–10 cm correlated with annualized crop residue and grain yield (r = 0.94–0.97, *P* ≤ 0.05). Increased crop residue returned to the soil increased soil C stock with CW and W-P and N stock with W-P, but removal of aboveground crop biomass for hay decreased stocks with W-B-P. Increased soil N stock had a beneficial effect on crop grain yield.

## 1 Introduction

It has been known that several decades of conventional tillage with crop-fallow system reduced crop yields and soil organic matter by 30 to 50% from their original levels due to mineralization of organic matter and reduction in the amount of crop residue returned to the soil under dryland cropping systems in the northern Great Plains, USA (Peterson et al., 1998; Aase and Pikul, 1995; Sainju et al., 2017). Using improved management practices, such as no-tillage (NT), continuous cropping, and increased cropping intensity, decline in crop yields and soil organic matter can be reversed and become sustainable for the semiarid dryland cropping systems in this region (Halvorson et al., 2002; Sherrod et al., 2003; Sainju et al., 2017; Engel et al., 2017). Besides conserving soil C and N due to reduced soil disturbance, the NT system can increase soil water content and crop yields compared with conventional tillage (CT) system, which resulted in more producers adapting NT continuous cropping system than CT crop-fallow in the northern Great Plains (Aase and Pikul, 1995; Lenssen et al., 2007; Sainju et al., 2017).

Numerous studies (Peterson et al., 1998; Halvorson et al., 2002: Sainju et al., 2007, Sainju et al., 2017) have shown the beneficial effect of NT and diversified crop rotations on soil C and N stocks compared with CT and monocropping. Increasing cropping intensity increased soil organic C (SOC) and total N (STN) at 0–20 cm in the NT system after 12 yr (Sherrod et al., 2003). The NT can sequester C at 570 ± 140 kg C ha^−1^ yr^−1^, reaching equilibrium in 15 to 20 yr compared with CT and enhanced crop rotation can sequester C at 200 ± 120 kg C ha^−1^ yr^−1^, reaching equilibrium in 40 to 60 yr compared with monocropping (West and Post, 2002). Sainju et al. (2007) reported that NT with continuous cropping reduced the decline of SOC and STN from their original levels under grasslands compared with CT with crop-fallow. Enhancing C and N stocks can also improve soil structure and soil water-nutrient-crop productivity relationships (Bauer and Black, 1994; Sainju et al., 2007, Sainju et al., 2017) and mitigate emissions of greenhouse gases, such as CO_2_ and N_2_O, in the atmosphere (Paustian et al., 1997; Lal et al., 1999).

Crop rotation is an effective management of controlling weeds, diseases, and pests (Miller et al., 2002; Tanaka et al., 2002), reducing the risk of crop failure, farm inputs, and duration of fallow, and improving economic and environmental sustainability of dryland cropping systems (Matson et al., 1997; Gregory et al., 2002). Diversified crop rotations can efficiently use water and N compared with monocropping (Miller et al., 2002; Lenssen et al., 2007). For instance, rotating nonlegume crops, such as wheat and barley, with legume crops, such as pea, can efficiently utilize soil water in dryland cropping systems, because pea uses less water than wheat and barley, resulting in more water available for succeeding crops in the rotation (Miller et al., 2002; Lenssen et al., 2007). Pea residue also supplies more N to succeeding crops because of its higher N concentration than wheat or barley residue, thereby reducing N fertilization rates (Miller et al., 2002; Sainju et al., 2007, Sainju et al., 2017). Both soil C and N stocks can be influenced by the quality and quantity of residue returned to the soil from crops involved in the rotation (Kuo et al., 1997; Campbell et al., 2000; Sainju et al., 2007).

Another approach to control weeds and pests includes improved cultural practices where seeding rates, planting and harvest dates, and methods of fertilization are varied so that weeds compete with crops in water and nutrient availability and timing of growths for hosts and pests are altered. This results in favorable environment for crop growth. Increased seeding rate increases weed and crop competition, banded fertilization limits nutrient availability to weeds, delayed planting after late application of preplant herbicide kills weed seedlings, and tall stubble increases soil water content by catching more snow and reduces light penetration into the ground which reduces weed germination (Strydhorst et al., 2008; Nichols et al., 2015). Some researchers (Anderson, 2000; Entz et al., 2002; Strydhorst et al., 2008) reported that increased crop seeding rates, banded fertilization method, delayed planting and harvest dates, increased retention of crop residue at the soil surface, and inclusion of forages in the crop rotation reduced weed growth compared with conventional seeding rates, normal planting and harvest dates, broadcast fertilization, reduced residue retention, and monocropping. The long-term effects of such diversified crop rotations and cultural practices on soil C and N stocks under dryland cropping systems is lacking.

Soils under dryland cropping systems also contain high inorganic C (SIC), especially in subsoil layers (Cihacek and Ulmer, 2002; Monger, 2002; Sainju et al., 2007). Management practices can influence both SIC and soil total C (STC as sum of SIC and SOC), besides SOC (Cihacek and Ulmer, 2002; Monger, 2002; Sainju et al., 2007, Sainju et al., 2017). For example, CT can increase SIC compared with NT (Monger, 2002) and continuous application of NH_4_-based N fertilizers can increase soil acidity which solubilize SIC in the surface soil and translocate it to the subsoil layers (Mikahilova and Post, 2006). Similarly, crop rotations and cultural practices had variable effect on STC (Sainju et al., 2017). As a result, we decided to evaluate the effect of management practices on STC instead of SOC. If management practices can influence STC, then changes in STC can be used as a better predictor of total C sequestration than SOC (Sainju et al., 2017). This can also reduce the cost of analyzing soil samples by measuring only for STC instead of the double analysis required for SOC and SIC to measure soil C sequestration.

Our objectives were to: (1) quantify the long-term effect of tillage, crop rotation, and cultural practice on annualized crop residue returned to the soil and grain yield from 2004 to 2011 under dryland cropping systems in the northern Great Plains, USA, (2) evaluate their effect on STC and STN at the 0–120 cm depth in 2011, and (3) relate STC and STN with annualized crop residue and grain yield. We hypothesized that NT with diversified crop rotation and the improved cultural practice would increase soil C and N stocks and sustain crop yields compared to CT with monocropping and the traditional practice.

## 2 Materials and methods

### 2.1 Site and treatment descriptions

The 8-yr experiment (2004–2011) was conducted at a dryland farm site, 8 km northwest of Sidney (47° 46′N, 104° 16′W; elevation 690 m), Montana, USA. The experimental site has mean monthly air temperature ranging from −8 °C in January to 23 °C in July and August and mean annual precipitation (105-yr average) of 340 mm, 80% of which is received during the crop growing season (April–October). The soil at the site was Williams loam (fine-loamy, mixed, superactive, frigid, Typic Argiustolls) and had 350 g kg^−1^ sand, 325 g kg^−1^ silt, 325 g kg^−1^ clay, and 6.1 pH at the 0–20 cm depth. The STC at 0–5 cm prior to the initiation of the experiment in April 2004 was 7.29 Mg C ha^−1^, STN 0.76 Mg N ha^−1^, and bulk density 1.35 Mg m^−3^. At 5–20 cm, STC was 20.08 Mg C ha^−1^, STN 1.96 Mg N ha^−1^, and bulk density 1.44 Mg m^−3^. The cropping system prior to the experiment initiation was spring wheat-fallow under CT.

Treatments were two tillage practices (NT and CT) as the main plot and a factorial combination of four crop rotations (continuous spring wheat [CW], spring wheat-pea [W-P], spring wheat-barley hay-pea [W-B-P], and spring wheat-barley hay-corn-pea [W-B-C-P]) and two cultural practices (traditional and improved) as the sub-plot variable arranged in a randomized block design with three replications. The CW was a 1-yr rotation with one crop phase (spring wheat); W-P, a 2-yr rotation with two phases (spring wheat and pea); W-B-P, a 3-yr rotation with three phases (spring wheat, barley hay, and pea), and W-B-C-P, a 4-yr rotation with four phases (spring wheat, barley hay, corn, and pea). Every phase of the crop rotation was present in each year. The sequence of crops in each rotation from 2004 to 2011 is shown in Table 1. The description of cultural practices used for each crop in the rotation is shown in Table 2. Plots under NT were left undisturbed, except for applying fertilizers and planting crops in rows. Plots under CT were tilled one to two times a year with a field cultivator to a depth of 7–8 cm for seedbed preparation and weed control. The size of the main plot was 36.6 × 12.2 m and the split plot was 12.2 × 12.2 m.

**Table 1:** Description of crops in the crop rotation employed in all tillage and cultural practice treatments from 2004 to 2011.

Crop rotation^a^	No. of plot	2004	2005	2006	2007	2008	2009	2010	2011
CW	1	Wheat	Wheat	Wheat	Wheat	Wheat	Wheat	Wheat	Wheat
W-P	1	Wheat	Pea	Wheat	Pea	Wheat	Pea	Wheat	Pea
	2	Pea	Wheat	Pea	Wheat	Pea	Wheat	Pea	Wheat
W-B-P	1	Wheat	Barley hay	Pea	Wheat	Barley hay	Pea	Wheat	Barley hay
	2	Barley hay	Pea	Wheat	Barley hay	Pea	Wheat	Barley hay	Pea
	3	Pea	Wheat	Barley hay	Pea	Wheat	Barley hay	Pea	Wheat
W-B-C-P	1	Wheat	Barley hay	Corn	Pea	Wheat	Barley hay	Corn	Pea
	2	Barley hay	Corn	Pea	Wheat	Barley hay	Corn	Pea	Wheat
	3	Corn	Pea	Wheat	Barley hay	Corn	Pea	Wheat	Barley hay
	4	Pea	Wheat	Barley hay	Corn	Pea	Wheat	Barley hay	Corn

^a^ Crop rotations are CW, continuous spring wheat; W-P, spring wheat-pea; W-B-P, spring wheat-barley hay-pea; and W-B-C-P, spring wheat-barley hay-corn-pea.

**Table 2:** Description of cultural practices (traditional and improved) used for crops in the rotation employed in all tillage practices.

Crop	Cultural practice	Seeding rate (million seeds ha^−1^)	N fertilization at planting	N fertilization rate (kg N ha^−1^)	Planting date	Stubble height (cm)
Spring wheat	Traditional	2.23	Broadcast	101	Early April	20
	Improved	2.98	Banded	101	Early May	30
Pea	Traditional	0.60	Broadcast	6	Early April	5
	Improved	0.92	Banded	6	Early April	5
Barley hay	Traditional	2.23	Broadcast	67	Late April	5
	Improved	2.98	Banded	67	Late April	5
Corn	Traditional	0.04	Broadcast	78	Early May	5
	Improved	0.05	Broadcast	78	Early May	5

### 2.2 Management of crops

At planting in early April to early May, 2004 to 2011, P fertilizer as mono-ammonium phosphate (11% N, 23% P) at 56 kg P ha^−1^ and K fertilizer as muriate of potash (60% K) at 48 kg K ha^−1^ were banded to all crops to a depth of 5 cm below and 5 cm away from seeds. At the same time, N fertilizer as urea (46% N) and monoammonium phosphate were applied to spring wheat, barley hay, and corn at recommended rates shown in Table 2. Pea received N fertilizer at 6 kg N ha^−1^ from monoammonium phosphate. Recommended N rates included both actual N rate and soil residual N which was determined as soil NO_3_-N content to a depth of 60 cm collected after crop harvest in the autumn of the previous year. Actual N rate, therefore, is determined by deducting soil residual N from the recommended N rate. This was done to avoid excessive application of N fertilizer. The N fertilizer was either broadcast in the traditional cultural practice or banded in the improved practice, except for corn where N fertilizer was broadcast in both cultural practices (Table 2). Using a low disturbance no-till drill, spring wheat (cv. Reeder) was planted from early April to early May, pea (cv. Majoret) in early April, and barley hay (cv. Haybet) in late April at a spacing of 20 cm, while corn (cv. 39T67-RR from 2004–2008 and Pioneer 39D95-RR from 2009–2011) was planted in early May at a spacing of 60 cm. Appropriate herbicides and pesticides were applied for each crop at preplanting, during growth, and at postharvest (Lenssen et al., 2014). No irrigation was applied.

In late June and early July, barley hay was harvested by cutting aboveground biomass from an area of 1.5 × 12.0 m with a self-propelled mower-conditioner and round baler after determining biomass yield from two 0.5 m^2^ areas outside yield rows on oven-dried (65 °C for 3 d) basis. In August, total biomass (grains + stems + leaves) yield of spring wheat and pea was determined from two 0.5 m^2^ areas as above and grain yield (oven-dried basis) was determined by harvesting grains from a swath of 1.5 × 12.0 m using a combine harvester. In October, corn total biomass and grain yields were determined from areas as described above. Crop residue of spring wheat, pea, and corn, was determined by deducting grain yield from total biomass yield. After grain harvest, crop residue of spring wheat, pea, and corn were returned to the soil. Annualized crop residue or grain yield for a crop rotation was calculated by dividing the sum of crop residue or grain yield of all crops by the number of crops in the rotation in a year. Because aboveground biomass was removed for hay and grain yield did not exist in barley, crop residue returned to the soil and grain yield of barley were considered zero in the calculation of annualized crop residue and grain yield in W-B-P and W-B-C-P.

### 2.3 Soil sampling and analysis

After final crop harvest in late October 2011, soil samples were collected from the 0–120 cm depth using a tractor-mounted hydraulic probe (3.5 cm inside diameter) after clearing the surface crop residue in all plots. Samples were collected from five places covering locations within and between crop rows in the central areas of each plot, separated into 0–5, 5–10, 10–20, 20–40, 40–60, 60–90, and 90–120 cm segments to represent each depth, composited by depth, placed in plastic bags in a cooler, and transported to the laboratory. About 10 g soil from each plot and depth was oven-dried at 110 °C for 24 hr to determine dry weight, from which the bulk density was determined by dividing the weight of oven-dried soil by the volume of the core. Rest of the soil was air-dried, ground, and sieved to 2 mm for determining STC and STN concentrations.

The STC and STN concentrations (g C or N kg^−1^) in all soil samples were determined by using a high induction furnace C and N analyzer (Elementar, Mt. Laurel, New Jersey, USA) after grinding the samples to ≪0.5 mm. The STC and STN contents (Mg C or N ha^−1^) at 0–5, 5–10, 10–20, 20–40, 40–60, 60–90, and 90–120 cm depths were calculated by multiplying their concentrations by the bulk density and the thickness of the soil layer. Because the bulk density was not affected by treatments, average bulk density values of 1.13, 1.35, 1.37, 1.49, 1.55, 1.61, and 1.63 Mg m^−3^ at 0–5, 5–10, 10–20, 20–40, 40–60, 60–90, and 90–120 cm, respectively, across treatments were used for converting STC and STN concentrations into contents. Because soil mass was not significantly different among treatments due to nonsignificant differences in the bulk density, the equivalent soil mass method as proposed by Lee et al. (2009) to convert STC and STN concentrations into contents was not used. Total STC and STN contents at 0–120 cm were determined by summing the contents from individual depth layers. The STC or STN content for a crop rotation was calculated by dividing total STC or STN content under all crops by the number of crops in the rotation.

### 2.4 Statistical analysis of data

Data for annualized crop biomass and grain yields were analyzed using the MIXED procedure of SAS (Littell et al., 1996). Tillage, crop rotation, cultural practice, year and their interactions were considered as fixed effects and replication and tillage × replication as random effects. Similarly, data for STC and STN contents at a depth were analyzed using the MIXED procedure as above where tillage, crop rotation, cultural practice, and their interactions were considered as fixed effects and replication and tillage × replication as random effects. Means were separated by using the least square means test when treatments and interactions were significant (Littell et al., 1996). Statistical significance was evaluated at *P* ≤ 0.05, unless otherwise stated.

## 3 Results and discussion

### 3.1 Crop residue

Annualized crop residue returned to the soil after grain harvest, varied with crop rotations and years, with significant interactions for crop rotation × year and cultural practice × year (Table 3). Averaged across tillage and cultural practices, crop residue was greater with CW or W-P than W-B-P or W-B-C-P in 2004, 2005, 2006, 2007, and 2010 (Table 4). In 2008, crop residue was greater with W-B-C-P than CW, but was lower with W-B-P than other crop rotations in 2009. Averaged across tillage and crop rotations, crop residue was greater in the improved than the traditional practice in 2006 and 2009, but the trend reversed in 2005 and 2007. Averaged across tillage, cultural practices, and years, crop residue was greater with CW and W-P than other crop rotations (Table 3). Averaged across treatments, crop residue was lower in 2008 than other years. Tillage and its interaction with other treatments had no effect crop residue.

**Table 3:** Annualized crop residue (stems + leaves) returned to the soil and grain yield as influenced by crop rotation, cultural practice, and year.

Crop rotation^a^	Cultural practice^b^	Year	Crop residue (Mg ha^−1^)	Grain yield (Mg ha^−1^)
CW			3.92a^c^	2.14a
W-P			3.86a	2.10a
W-B-P			2.68c	1.51c
W-B-C-P			3.36b	1.84b
				
	Traditional		3.31	1.92a
	Improved		3.28	1.71b
				
		2004	4.64a	1.89b
		2005	3.68c	2.34a
		2006	4.46ab	1.72bc
		2007	4.28b	1.92b
		2008	1.50f	0.65d
		2009	3.09d	2.18ab
		2010	3.94c	2.38a
		2011	2.02e	1.57c
Significance				
Tillage (T)			NS	NS
Crop rotation (C)			***	***
T × C			NS	NS
Cultural practice (P)			NS	***
T × P			NS	NS
C × P			NS	NS
T × C × P			NS	NS
Year (Y)			***	***
T × Y			NS	NS
C × Y			***	***
T × C × Y			NS	NS
P × Y			*	NS
T × P × Y			NS	NS
C × P × Y			NS	NS
T × C × P × Y			NS	NS

*Significant at *P* = 0.05.

**Significant at *P* = 0.001; NS, not significant.

^a^ Crop rotations are CW, continuous spring wheat; W-P, spring wheat-pea; W-B-P, spring wheat-barley hay-pea; and W-B-C-P, spring wheat-barley hay-corn-pea.

^b^ See Table 1 for description of the cultural practice.

^c^ Numbers followed by different letters within a column are significantly different at *P* = 0.05 by the least square means test.

**Table 4:** Interactions between crop rotation, cultural practice, and year on annualized crop residue (stems + leaves) returned to the soil and grain yield.

Crop rotation^a^	Cultural practice^b^	Year												
		2004	2005		2006		2007		2008		2009		2010	2011
		Crop residue (Mg ha^−1^)												
CW		5.47a^c^		3.76b		5.67a		5.23a		1.27b		3.57a	4.01ab	2.41
W-P		5.06a		4.19a		4.78b		4.74ab		1.63ab		3.15a	4.87a	2.44
W-B-P		3.69b		2.96c		3.51c		3.35b		1.35ab		2.27b	3.97ab	1.56
W-B-C-P		4.73ab		3.92ab		3.57c		3.96b		1.77a		3.19a	3.69b	1.78
									
	Traditional	4.36		3.80a		3.80b		4.39a		1.63		2.65b	3.87	2.03
	Improved	4.50		3.48b		4.30a		3.65b		1.52		3.31a	3.74	1.77
		Grain yield (Mg ha^−1^)												
CW		2.99a	2.55		1.80a		2.32a		0.36b		2.52a		2.55a	2.07a
W-P		2.57a	2.45		1.92a		2.45a		0.76a		2.35a		2.76a	1.52ab
W-B-P		1.62b	2.10		1.37b		1.65b		0.68a		1.56b		1.86b	1.28b
W-B-C-P		1.51b	2.33		1.81a		1.78b		0.67a		2.97a		2.53a	1.63ab

^a^ Crop rotations are CW, continuous spring wheat; W-P, spring wheat-pea; W-B-P, spring wheat-barley hay-pea; and W-B-C-P, spring wheat-barley hay-corn-pea.

^b^ See Table 1 for description of the cultural practice.

^c^ Numbers followed by different letters within a column in a set are significantly different at *P* = 0.05 by the least square means test.

The greater crop residue with CW and W-P than other crop rotations in most years was due to enhanced performance of spring wheat and pea under dryland cropping systems in the semiarid region in the northern Great Plains. Although barley hay also performed well, removal of its aboveground biomass for hay reduced crop residue returned to the soil with W-B-P. This, along with poor performance of corn under dryland condition in some years resulted in similar or lower crop residue with W-B-C-P than CW and W-P. However, corn produced greater crop residue than spring wheat and pea, resulting in greater crop residue with W-B-C-P than CW in 2008 when the growing season precipitation was lower than other years and the 68-yr average (Table 5). Growing season (April–November) precipitation was 185 mm in 2008 compared with 217 to 397 mm in other years and the 68-yr average (Table 5), with May and June being drier than other months. The different trends in crop residue between traditional and improved cultural practices in 2005, 2006, 2007, and 2009 appeared to be a result of variations in monthly precipitation patterns. Increased precipitation in April and May, 2005 and 2007 (Table 5) promoted growth of early planted spring wheat, thereby increasing crop residue in the traditional compared with the improved practice in those years. The reverse was true in 2006 and 2009 when monthly precipitation was lower in April and May. The lower crop residue in 2008 than other years was due to decreased precipitation during the crop growing season which reduced crop growth. As biomass residue of all crops, except barley hay, was returned to the soil after grain harvest, variations in crop residue among treatments and years influenced STC and STN, as discussed below.

**Table 5:** Total annual and the growing season (April to November) precipitation from 2004 to 2011 at the experimental site.

Month	2004	2005	2006	2007	2008	2009	2010	2011	68-yr average
		Precipitation (mm)							
April	7	2	80	21	11	39	29	39	29
May	53	83	44	128	28	8	142	146	50
June	45	115	55	49	32	56	71	24	72
July	31	36	30	21	32	70	51	68	54
August	15	19	36	8	23	38	56	15	37
September	36	2	67	19	22	13	57	20	34
October	27	26	10	9	24	17	17	9	25
November	3	18	1	0	13	1	16	1	12
April-November	217	301	322	253	185	242	397	322	311
January-December	284	324	339	280	189	282	415	347	357

### 3.2 Crop grain yield

Annualized crop grain yield varied with crop rotations, cultural practices, and years, with a significant interaction for crop rotation × year (Table 3). Averaged across tillage and cultural practices, grain yield was greater with CW and W-P than other crop rotations in 2004 and 2007 (Table 4). In 2006, 2009, and 2010, grain yield was lower with W-B-P than other crop rotations. Grain yield was lower with CW than other crop rotations in 2008, but was greater with CW than W-B-P in 2011. Averaged across tillage, cultural practices, and years, grain yield was greater with CW and W-P than other crop rotations, a case similar to that observed for biomass yield (Table 3). Averaged across tillage, crop rotations, and years, grain yield was greater in the traditional than the improved practice. Averaged across treatments, grain yield was lower in 2008 than other years.

As with crop residue, greater grain yield with CW and W-P than other crop rotations in most years was due to increased spring wheat and pea yields. Absence of grain in barley hay reduced grain yield with W-B-P in all years, except in 2008. Lower growing season precipitation reduced spring wheat grain yield, reducing annualized grain yield with CW in 2008. Greater grain yield with W-B-C-P in 2006, 2008, 2009, and 2010 was due to increased corn yield, probably as result of efficient water and N use and/or reduced incidences of weeds and pests. Several researchers (Miller et al., 2002; Lenssen et al., 2007, Lenssen et al., 2014; Sainju et al., 2017) have reported greater wheat and barley yields with crop rotation compared with monocropping in the northern Great Plains. Efficient water use due to lower seeding rate and early planting also likely increased grain yield with the traditional than the improved cultural practice, as soil drying in the late planting can reduce crop growth and therefore grain yield if there was no precipitation during this period (Lenssen et al., 2014). Similar to crop residue, lower grain yield in 2008 than other years was due to reduced precipitation that resulted in lower available soil water content (Lenssen et al., 2014).

### 3.3 Soil total carbon

The STC at 5–10 and 10–20 cm varied with crop rotations (Table 6). Interactions were significant for tillage × crop rotation at 10–20 cm and tillage × crop rotation × cultural practice at 90–120 cm.

**Table 6:** Soil total C (STC) at the 0–120 cm depth as affected by crop rotation.

Crop rotation^a^	STC (Mg C ha^−1^)							
	0-5 cm	5-10 cm	10–20 cm	20–40 cm	40–60 cm	60–90 cm	90–120 cm	0–120 cm
CW	7.8	8.3a^b^	13.7a	61.7	91.2	130.0	122.1	434.9
W-P	7.3	7.8ab	13.2ab	66.2	96.8	130.0	119.8	439.5
W-B-P	7.2	7.6b	12.6b	62.0	96.7	136.4	117.2	433.4
W-B-C-P	7.4	7.8ab	12.8b	58.3	96.5	133.5	120.0	436.3
Significance								
Tillage (T)	NS	NS	NS	NS	NS	NS	NS	NS
Crop rotation (C)	NS	*	*	NS	NS	NS	NS	NS
T × C	NS	NS	*	NS	NS	NS	NS	NS
Cultural practice (P)	NS	NS	NS	NS	NS	NS	NS	NS
T × P	NS	NS	NS	NS	NS	NS	NS	NS
C × P	NS	NS	NS	NS	NS	NS	NS	NS
T × C × P	NS	NS	NS	NS	NS	NS	*	NS

*Significant at *P* = 0.05; NS, not significant.

^a^ Crop rotations are CW, continuous wheat; W-P, spring wheat-pea; W-B-P, spring wheat-barley hay-pea; and W-B-C-P, spring wheat-barley hay-corn-pea.

^b^ Numbers followed by different letters within a column are significantly different at *P* = 0.05 by the least square means test.

Averaged across cultural practices, STC at 10–20 cm was greater with W-P than W-B-C-P in CT and greater with CW than other crop rotations in NT (Table 7). The STC at 10–20 cm was greater in NT than CT with CW. At 90–120 cm, STC was greater with CW than W-B-P in CT with the improved practice and in NT with the traditional practice. With CW, STC at 90–120 cm was also greater in NT with the traditional practice than CT with the traditional practice and NT with the improved practice. Averaged across tillage and cultural practices, STC at 5–10 cm was greater with CW than W-B-P and at 10–20 cm was greater with CW than W-B-P and W-B-C-P (Table 6). The STC at all depths was not correlated with annualized crop biomass and grain yields (Table 8).

**Table 7:** Interactions between tillage, crop rotation, and cultural practice on soil total C (STC) at 10–20 and 90–120 cm depths.

Tillage^a^	Cultural practice^b^	Crop rotation^c^			
		CW	W-P	W-B-P	W-B-C-P
		STC at 10–20 cm (Mg C ha^−1^)			
CT		12.7b^d^AB^e^	13.5A	12.5AB	12.3B
NT		14.7aA	12.7B	12.6B	13.3B
		STC at 90–120 cm (Mg C ha^−1^)			
CT	Traditional	112.7b	117.3	117.6	117.5
	Improved	128.2abA	113.8AB	112.3B	118.2AB
NT	Traditional	130.8aA	120.3AB	114.4B	117.1AB
	Improved	116.9b	127.8	124.6	127.1

^a^ Tillage are CT, conventional tillage; and NT no-tillage.

^b^ See Table 1 for description of the cultural practice.

^c^ Crop rotations are CW, continuous spring wheat; W-P, spring wheat-pea; W-B-P, spring wheat-barley hay-pea; and W-B-C-P, spring wheat-barley hay-corn-pea.

^d^ Numbers followed by different lowercase letters within a column in a set are significantly different at *P* = 0.05 by the least square means test.

^e^ Numbers followed by different uppercase letters within a row in a set are significantly different at *P* = 0.05 by the least square means test.

**Table 8:** Correlation analysis between soil total C (STC) and total N (STN) at 0–10, 0–20, and 0–120 cm depths and mean annualized crop residue returned to the soil and grain yield (n = 4).

Soil parameter	Soil depth	Crop residue (Mg ha^−1^)	Grain yield (Mg ha^−1^)
STC (Mg C ha^−1^)	0–10 cm	0.71	0.72
	0–20 cm	0.81	0.82
	0–120 cm	0.52	0.53
STN (Mg N ha^−1^)	0–10 cm	0.97*	0.94*
	0–20 cm	0.86	0.86
	0–120 cm	0.27	0.27

*Significant at *P* = 0.05.

The greater crop residue returned to the soil likely increased STC at 10–20 and 90–120 cm with CW and W-P than other crop rotations in NT and CT with traditional and improved cultural practices. Annualized crop residue returned to the soil was greater with CW and W-P than other crop rotations in most years (Table 3 and Table 4). Several researchers (Sherrod et al., 2003; Sainju et al., 2017; Engel et al., 2017) also reported that increased crop residue returned to the soil increased STC and SOC to a depth of 120 cm in the northern Great Plains. The greater STC at 10–20 cm with W-P in CT appeared to be related to residue incorporation into the soil and with CW in NT to residue quality. It is likely that the incorporation of residue with higher N concentration, such as pea with average N concentration of 19.1 g N kg^−1^ across treatments compared with 15.1 g N kg^−1^ in spring wheat, due to tillage increased turnover rate of plant C into soil C, thereby increasing SOC with W-P in CT. Sainju (2014) and Engel et al. (2017) also observed greater SOC with malt barley-pea or spring wheat-pea than continuous malt barley or spring wheat, respectively, in CT. Residues with higher N concentration decompose more rapidly than residues with lower N concentration (Kuo et al., 1997). The CT can increase SIC compared with NT (Monger, 2002). As STC is composed of both SOC and SIC, the greater STC with W-P in CT could be a result of increases in both SOC and SIC.

The reverse can be true for greater STC at 10–20 cm with CW than other crop rotations in NT where spring wheat biomass residue with intermediate N concentration was accumulated at the soil surface in NT. It is likely that the undisturbed soil condition reduced C mineralization from crop residue and soil organic matter, thereby increasing STC with CW in NT. This is also supported by greater STC at 10–20 cm in NT than CT with CW (Table 7). Undisturbed soil condition also may have increased STC at 90–120 cm with CW in NT with the traditional practice. Greater SOC or STC in NT than CT or greater with continuous nonlegume than a mixture of legume-nonlegume-oilseed crops in NT under dryland cropping systems in the northern Great Plains, USA have been reported (Halvorson et al., 2002; West and Post, 2001; Sainju 2014, Engel et al., 2017; Sainju et al., 2017).

The lower STC at 5–10 and 10–20 cm with W-B-P than other crop rotations (Table 6) could be a result of removal of aboveground barley biomass for hay. Because the pH of the soil at 0–20 cm was 6.1, the SIC content can be negligible and most of STC can be considered as SOC at this layer (Mulvaney, 1996). Some researchers (Kuo and Jellum, 2002; Blanco-Canqui, 2010) have reported that removal of aboveground crop residue reduces C input to the soil and therefore SOC compared with non-removal of the residue.

The original level of STC at 0–20 cm at the initiation of experiment in 2004 was 27.4 Mg C ha^−1^. The STC at 0–20 cm after 8 yr of the experiment was 29.8, 28.8, 27.4, and 28.0 Mg C ha^−1^ with CW, W-P, W-C-B, and W-B-C-P respectively (Table 6). This suggests that C was sequestered at 300, 175, 0, and 75 kg C ha^−1^ yr^−1^ with CW, W-P, W-C-B, and W-B-C-P, respectively. Several researchers (Halvorson et al., 2002; Sainju et al., 2007; VandenBygaart et al., 2011) have found C sequestration rates at 0–20 cm ranging from 230 to 400 kg C ha^−1^ yr^−1^ with continuous nonlegume cropping compared with crop-fallow under dryland cropping systems in the northern Great Plains, USA. Similarly, Sainju et al. (2017) reported greater C sequestration rate at 0–125 cm with continuous nonlegume cropping than a mixture of legume-nonlegume-oilseed crop rotation. Our results suggest that crop rotation can have a larger impact on soil C sequestration than tillage and cultural practice and that continuous nonlegume monocropping can sequester C at a greater rate than crop rotation containing legume and nonlegume crops under dryland cropping systems in the semiarid regions of the northern Great Plains, USA. Removal of aboveground biomass for hay, however, can dramatically reduce C sequestration rate.

### 3.4 Soil total nitrogen

The STN varied with tillage at 0–5 and 5–10 cm and with crop rotations at 20–40, 60–90, and 0–120 cm (Table 9). Significant interactions occurred for tillage × crop rotation at 0–5 and 20–40 cm, for tillage × cultural practice at 5–10 cm, and for crop rotation × cultural practice at 60–90 cm.

**Table 9:** Soil total N (STN) at the 0–120 cm depth as affected by tillage and crop rotation.

Crop rotation^a^	Tillage^b^	STN (Mg N ha^−1^)							
		0-5 cm	5-10 cm	10–20 cm	20–40 cm	40–60 cm	60–90 cm	90–120 cm	0–120 cm
CW		0.82	0.91	1.46	2.34b^c^	2.11	2.29b	2.11	12.03b
W-P		0.85	0.90	1.53	2.66a	2.24	2.55a	2.23	12.96a
W-B-P		0.79	0.86	1.44	2.43ab	2.17	2.35b	2.22	12.17b
W-B-C-P		0.81	0.88	1.47	2.54a	2.26	2.51a	2.10	12.62ab
									
	CT	0.79b	0.86b	1.41	2.40	2.12	2.37	2.14	12.09
	NT	0.84a	0.91a	1.54	2.53	2.27	2.48	2.19	12.80
Significance									
Tillage (T)		*	*	NS	NS	NS	NS	NS	NS
Crop rotation (C)		NS	NS	NS	**	NS	*	NS	*
T × C		*	NS	NS	*	NS	NS	NS	NS
Cultural practice (P)		NS	NS	NS	NS	NS	NS	NS	NS
T × P		NS	*	NS	NS	NS	NS	NS	NS
C × P		NS	NS	NS	NS	NS	*	NS	NS
T × C × P		NS	NS	NS	NS	NS	NS	NS	NS

*Significant at *P* = 0.05.

**Significant at *P* = 0.01; NS, not significant.

^a^ Crop rotations are CW, continuous spring wheat; W-P, spring wheat-pea; W-B-P, spring wheat-barley hay-pea; and W-B-C-P, spring wheat-barley hay-corn-pea.

^b^ Tillage are CT, conventional tillage; and NT, no-tillage.

^c^ Numbers followed by different letters within a column in a set are significantly different at *P* = 0.05 by the least square means test.

Averaged across cultural practices, STN at 0–5 cm was greater with W-P than other crop rotations in NT and greater in NT than CT with W-P (Table 10). At 20–40 cm, STN was greater with W-P than CW and W-B-P in CT and greater with W-P and W-B-C-P than W-B-P in NT. The STN at 20–40 cm was greater also in NT than CT with CW. Averaged across crop rotations, STN at 5–10 cm was greater with the improved than the traditional cultural practice in NT and greater in NT than CT with the improved practice. Averaged across tillage, STN at 60–90 cm was greater with W-P and W-B-C-P than CW and W-B-P in the improved practice and greater in the traditional than the improved practice with CW. Averaged across tillage and cultural practices, STN at 20–40 and 60–90 cm was greater with W-P and W-B-C-P than CW or W-B-P and at 0–120 cm was greater with W-P than CW and W-B-P (Table 9). The STN at 0–10 cm was correlated with mean annualized crop biomass and grain yields across tillage, cultural practices, and years (Table 8).

**Table 10:** Interactions between tillage, crop rotation, and cultural practice on soil total N (STN) at 0–5, 5–10, 20–40, and 60–90 cm.

Crop rotation^a^	Cultural practice^b^	Tillage^c^	
		CT	NT
		STN at 0–5 cm (Mg N ha^−1^)	
CW		0.81	0.82b^d^
W-P		0.77B^e^	0.96aA
W-B-P		0.77	0.81b
W-B-C-P		0.80	0.83b
		STN at 5–10 cm (Mg N ha^−1^)	
	Traditional	0.87	0.88b
	Improved	0.86B	0.94aA
		STN at 20–40 cm (Mg N ha^−1^)	
CW		2.10cB	2.58abA
W-P		2.62a	2.70a
W-B-P		2.42b	2.44b
W-B-C-P		2.46ab	2.61a
		Cultural practice	
		Traditional	Improved
		STN at 60–90 cm (Mg N ha^−1^)	
CW		2.51A	2.04cB
W-P		2.47	2.64a
W-B-P		2.35	2.36b
W-B-C-P		2.41	2.61a

^a^ Crop rotations are CW, continuous spring wheat; W-P, spring wheat-pea; W-B-P, spring wheat-barley hay-pea; and W-B-C-P, spring wheat-barley hay-corn-pea.

^b^ See Table 1 for description of the cultural practice.

^c^ Tillage are CT, conventional tillage; and NT, no-tillage.

^d^ Numbers followed by different lowercase letters within a column in a set are significantly different at *P* = 0.05 by the least square means test.

^e^ Numbers followed by different uppercase letters within a row in a set are significantly different at *P* = 0.05 by the least square means test.

The greater STN at 0–5, 20–40, and 60–90 cm with W-P than CW and W-B-P was likely a result of increased crop residue N returned to the soil. Annualized crop residue N returned to the soil was 59, 66, 43, and 48 kg N ha^−1^ yr^−1^ with CW, W-P, W-B-P, and W-B-C-P, respectively. Although crop residue was greater with CW (Table 3), increased N concentration in pea residue (19.1 g N kg^−1^) compared with spring wheat residue (15.1 g N kg^−1^) increased the quantity of residue N returned to the soil and therefore increased STN with W-P. Greater STN at 20–40 and 60–90 cm was also observed with W-B-C-P in NT and the improved management practice whose reasons were not clear. Although we did not measure root biomass yield and N content, it may be possible that diversified crop rotation enhanced root growth and N input, thereby increasing STN with W-B-C-P in subsoil layers. Increased STN with crop rotations compared with monocropping due to enhanced N input has been known (Eck and Jones, 1992; Sherrod et al., 2003; Sainju et al., 2007).

As with STC, greater STN in NT than CT with W-P at 0–5 cm, with the improved cultural practice at 5–10 cm, and with CW at 20–40 cm may be a result of reduced N mineralization due to undisturbed soil condition. Increased STN in NT than CT under dryland cropping systems in the northern Great Plains has been known (Sainju et al., 2007; Engel et al., 2017). The greater STN at 5–10 cm with the improved than the traditional practice in NT was probably due to enhanced root growth and N input due to higher seeding rate and banded N fertilization. We did not measure root biomass yield, but crop residue varied with cultural practices in various years (Table 4), with no significant effect of cultural practice on crop residue (Table 3). The reasons for greater STN at 60–90 cm with the traditional than the improved practice with CW were not known.

The original STN at 0–20 cm at the initiation of the experiment in April 2004 was 2.72 Mg N ha^−1^. At the end of the experiment in 2011, STN at 0–20 cm was 3.19, 3.28, 3.09, and 3.16 Mg N ha^−1^ with CW, W-P, W-B-P, and W-B-C-P, respectively. This indicates that N was sequestered at 59, 70, 46, and 55 kg N ha^−1^ yr^−1^ with CW, W-P, W-B-P, and W-B-C-P, respectively. Similarly, STN at 0–20 cm at the end of the experiment was 3.06 and 3.29 Mg N ha^−1^ in NT and CT, respectively, suggesting corresponding N sequestration rates of 43 and 71 kg N ha^−1^ yr^−1^ in CT and NT. These data suggests that W-P and NT had higher N sequestration rates than other crop rotations and tillage practices. In dryland cropping systems in the northern great Plains, N sequestration rates vary from 40–52 kg N ha^−1^ yr^−1^ for crop rotations (Sainju et al., 2007; Sainju and Lenssen, 2011) and from −2 kg N ha^−1^ yr^−1^ in CT to 66 kg N in NT (Sainju et al., 2007).

The significant correlations between STN at 0–10 cm and annualized crop residue and grain yields, but not at 0–20 and 0–120 cm (Table 8), indicates that STN at the plow layer can be enhanced by crop residue N input and that increased STN can benefit dryland crop grain yield. Plots were tilled to a depth of 7–8 cm using field cultivator in CT, while NT plots were left undisturbed. The 0–20 cm layer represents the plow layer under dryland and irrigated cropping systems when moldboard plow is used for tillage. The 0–120 cm layer is the whole soil profile where STC and STN were evaluated. Although increased crop residue N input also increased STN at other depths, the plow layer appeared to be readily impacted by management practices for STN. The significant correlation between STN and grain yield was likely a result of enhanced soil water-nutrient-crop productivity relationship, as STN is a good indicator of soil organic matter (Bauer and Black, 1994; Sainju et al., 2007).

## 4 Conclusions

Eight years of tillage, crop rotation, and cultural practice had variable effect on annualized crop residue returned to the soil, grain yield, and STC and STN under dryland cropping systems in semiarid regions in the northern Great Plains, USA. Both crop residue and grain yields were greater with CW and W-P than other crop rotations in most years, but crop residue returned to the soil varied with cultural practices in various years. As a result, STC was greater with CW and W-P and STN was greater with W-P and W-B-C-P than other crop rotations in most soil depths, especially in NT and the improved cultural practice. Both STC and STN were also greater in NT than CT. Increased STN in the plow layer also benefited grain yield. No-tillage legume-nonlegume crop rotation can enhance soil C and N stocks and sustain crop yield compared with conventional tillage monocropping under dryland cropping systems, which supported part of our hypothesis. Removal of aboveground barley biomass for hay, however, reduced soil C and N stocks in the W-B-P rotation.

## Declarations

### Author contribution statement

Upendra M. Sainju: Conceived and designed the experiments; Analyzed and interpreted the data; Wrote the paper.

Andrew W. Lenssen: Performed the experiments.

Brett L. Allen, William B. Stevens, Jalal D. Jabro: Contributed reagents, materials, analysis tools or data.

### Funding statement

This research did not receive any specific grant from funding agencies in the public, commercial, or not-for-profit sectors.

### Competing interest statement

The authors declare no conflict of interest.

### Additional information

No additional information is available for this paper.
